# Unravelling the aromatic symphony: redirecting bifunctional mushroom synthases towards linalool monofunctionality

**DOI:** 10.1007/s44307-024-00056-2

**Published:** 2025-01-13

**Authors:** Rehka T, Fu Lin, Xixian Chen, Congqiang Zhang

**Affiliations:** Singapore Institute of Food and Biotechnology Innovation (SIFBI), Agency for Science, Technology and Research (A*STAR), Singapore, Republic of Singapore

**Keywords:** Terpene synthase, Linalool, Enzyme engineering, Synthetic biology, Biosynthesis

## Abstract

Enzymes are the cornerstone of biocatalysis, biosynthesis and synthetic biology. However, their applicability is often limited by low substrate selectivity. A prime example is the bifunctional linalool/nerolidol synthase (LNS) that can use both geranyl diphosphate (GPP) and farnesyl diphosphate (FPP) to produce linalool and nerolidol, respectively. This bifunctionality can lead to undesired byproducts in synthetic biology applications. To enhance enzyme specificity and create monofunctional linalool synthases, we modified amino acids in the loop between helices C and D of four bifunctional mushroom LNSs. Through these modifications, we successfully shifted the substrate preference of two LNSs (ApLNS from *Agrocybe pediades* and HsLNS from *Hypholoma sublateritium*) from FPP towards GPP. Although complete monofunctionality was not achieved, we significantly increased linalool yield by 13 times while minimizing nerolidol production to 1% of the wildtype HsLNS. Docking simulations revealed a substantial reduction in the FPP binding score compared to that of the wildtype. Molecular dynamics simulations suggested that Tyr300 in the apo HsLNS mutant has a greater tendency to adopt an inward orientation. Together with Met77, the inward-facing Tyr300 creates a steric barrier that prevents the longer FPP molecule from entering the substrate binding pocket, thereby greatly reducing its activity towards FPP. This study demonstrates the potential of enzyme engineering to design substrate-specific terpene synthases, which is a challenging task and few successful examples are available. The insights gained can inform future enzyme design efforts, including the development of artificial intelligence models.

**Supplementary Information:**

The online version contains supplementary material available at 10.1007/s44307-024-00056-2.

## Introduction

Terpenoids, particularly monoterpenes (C10) and sesquiterpenes (C15), are a major contributor to floral scent. Monoterpenes like linalool, limonene, myrcene, (E)-ocimene, and pinene, along with the sesquiterpene caryophyllene are among the most prevalent natural molecules in floral scents, found in over half of all seed plant families (Knudsen et al. 2006). Linalool, renowned for its pleasant floral aroma and low odour threshold (0.8–7.4 ppb), is a commercially significant molecule, with a global consumption exceeding 11,000 tons in 2018 (Zhang et al. 2021). However, the current supply in the market predominantly relies on fossil fuel-based chemical synthesis, a process that is unsustainable and generates substantial CO_2_ emissions. Biosynthesis of linalool using microbes from renewable carbon sources offers a promising alternative. To achieve this, a linalool synthase with a high activity, high specificity, and efficient heterologous expression in industrial microbes is essential. For many years, extensive research has focused on terpene synthases in flowering plants, with many plant linalool synthases (LSs) identified. However, plant LSs generally have poor expression, solubility and activity in microbes (Cao et al. 2017; Zhou et al. 2021). Consequently, plant LSs are not optimal for microbial linalool production (Ferraz et al. 2021). A study conducted 27 years ago reported that various mushrooms can produce diverse monoterpenes including linalool (Breheret et al. 1997). Particularly, linalool is found in the several edible and medicinal mushrooms like the tasty black poplar mushroom *Agrocybe aegerita* (recently renamed *Cyclocybe aegerita*) (Breheret et al. 1997), the model medicinal mushroom *Ganoderma lucidum* (lingzhi), lion's mane mushroom *Hericium erinaceus* (Pennerman et al. 2015). However, for over two decades, the responsible enzymes for linalool production in these fungi have remained elusive. To address this knowledge gap, our laboratory initiated a search for the LSs in these mushrooms. Through bioinformatics analysis and experiments, we identified the first known fungal linalool/nerolidol synthase (AaLNS) in *A. aegerita*. Building on this discovery, we further identified four additional novel mushroom LSs, ApLS and ApLNS from the common fieldcap *Agrocybe pediades*, GmLNS from the funeral bell mushroom *Galerina marginata*, and HsLNS from the brick cap mushroom *Hypholoma sublateritium* (Zhang et al. 2021).

Among the five fungal LSs studied, only ApLS demonstrated strict specificity for geranyl diphosphate (GPP), producing linalool exclusively. In contrast, the others accept both GPP and farnesyl diphosphate (FPP), yielding linalool (C10) and nerolidol (C15), respectively. To understand the molecular basis of this specificity, we compared the 3D structures of ApLNS and ApLS. We found that introducing the A59S and L60M mutations into ApLS enabled it to accept the larger FPP substrate, albeit with low nerolidol synthase activity (Zhang et al. 2021). Further analysis through segment swapping revealed that the 60–69 amino acid loop in ApLS plays a critical role in substrate specificity. The loop is situated between helix C and D, hence named the hCD loop. Replacing this loop with the corresponding sequence from ApLNS significantly increased nerolidol production in ApLS (T et al. 2023). Structural analysis of ApLS (PDB ID: 8GY0) elucidated that the Tyr299 orientation in ApLS was influenced by mutations in the hCD loop, particularly V61I. Tyr299, together with Ser184 and Met77, precludes the binding of larger substrates like FPP. In addition, the single amino acid substitutions like Y299A, Y299C, Y299G and Y299S not only widened the substrate scope of ApLS but also changed the cyclization and water incorporation of the final products. These mutants produced cyclized terpenes like α-, β-selinene and β-elemene instead of the linear terpene alcohol, linalool, for the wildtype ApLS (T et al. 2023).

To engineer monofunctional linalool synthases from the bifunctional mushroom LNSs, we replaced the loop amino acids of the four LNSs with those from ApLS and explored various combinations. Although complete monofunctionality was not achieved, we managed to boost the linalool yield up to 13 times while reducing nerolidol production by 99% compared to that of wildtype LNSs. This study serves an endeavor to design substrate-specific terpene synthases by targeted enzyme engineering. The insights gained can be used for future enzyme design, including the development of artificial intelligence models for protein engineering.

## Results and discussion

### Comparison of LNSs with ApLS

**Fig. 1:**
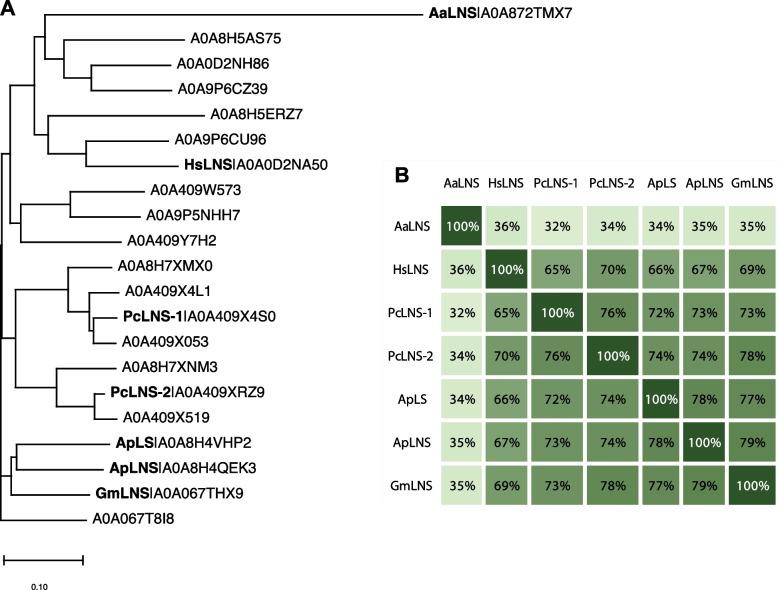
Alignment analysis of fungal linalool synthase (LS) homologues. **A** Polygenetic built by Neighbour-joining method without distance corrections. Here, the names of uncharacterized homologues are represented by their UniProt ID. **B** Percent identity matrix of characterized LS and LNSs

To identify homologous proteins, a BLAST sequence similarity search was conducted using ApLS in UniProt against a database of fungal genomes. A cutoff of 60% sequence identity and an E-value threshold of 1e-149 were applied, resulting in the identification of 20 homologous sequences (Fig. 1A). These homologs were distributed across six genera: *Agrocybe*, *Galerina*, *Gymnopilus*, *Hypholoma*, *Pholiota*, and *Psilocybe*. All the identified homologs belonged to the Basidiomycota, a group of higher fungi characterized by the production of fruiting bodies, commonly known as mushrooms. To confirm their functions, we cloned the two unknown homologues (UniProt ID: A0A409X4S0 and A0A409XRZ9) from *Psilocybe cyanescens* and characterized them in our engineered *E. coli* that supplies the terpene precursors GPP and FPP (Zhang et al. 2021). As expected, the two new enzymes from *P. cyanescens* also produced linalool and nerolidol, hence are renamed PcLNS-1 and PcLNS-2, respectively (Table 1 and Supplementary Fig. 1). To better quantify the specificity, we used the linalool and nerolidol percentage that are calculated by normalizing their titers (mg/L) with the titre sum of linalool and nerolidol of each strain overexpressing LNSs. Among the seven characterized enzymes, only ApLS produced 100% linalool (Table 1 and supplementary Fig. 2), GmLNS produced the highest amount of linalool (45%), and HsLNS produced the lowest amount of linalool (2%). In view of the highly conversed function of these homologues (Fig. 1A and 1B), we hypothesized that more LS/LNSs exist in higher fungi.

**Table 1:** Details of terpene synthases used

No	Enzymes	UniProt/ GenBank	Products	MW (kDa)	Organism	PDB
1	AaLNS	A0A872TMX7/ QOX59520.1	Nerolidol (96%), Linalool (4%)	43.4	*Agrocybe pediades*	
2	ApLS	A0A8H4VHP2/ KAF4609522.1	Linalool (100%)	39.7	*Cyclocybe aegerita* (Black poplar mushroom)	8GY0
3	ApLNS	A0A8H4QEK3^a^ / KAF4609589.1	Nerolidol (95%), Linalool (5%)	39.8	*Agrocybe pediades*	
4	GmLNS	A0A067THX9/ KDR79494.1	Nerolidol (55%), Linalool (45%)	39.4	*Galerina marginata*	
5	HsLNS	A0A0D2NA50/ KJA16009.1	Nerolidol (98%), Linalool (2%)	39.2	*Hypholoma sublateritium*	
6	PcLNS-1	A0A409X4S0/ PPQ85717.1	Nerolidol (97%), Linalool (3%)	39.9	*Psilocybe cyanescens*	
7	PcLNS-2	A0A409XRZ9/ PPQ93592.1	Nerolidol (91%), Linalool (9%)	39.2	*Psilocybe cyanescens*	

^a^There are 5 amino acid difference for the reference with our ApLNS sequence

### Engineering ApLNS for higher linalool activity by mutating the hCD loop region

**Fig. 2:**
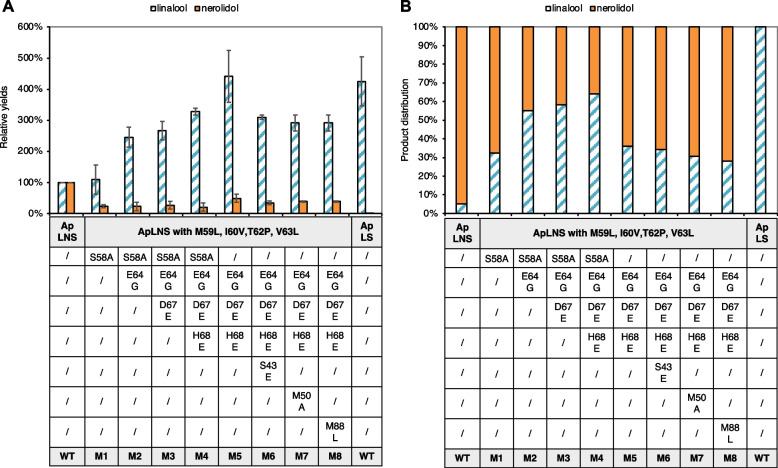
The effects of ApLNS mutation on linalool and nerolidol production. **A** Relative production of linalool and nerolidol. The values are normalized to that of the wildtype (WT) ApLNS. The standard deviation was calculated with four batches of GC–MS data. **B** Product distribution of linalool and nerolidol for ApLS, ApLNS and its mutants (M1-M8). Here, the ratio was calculated by linalool and nerolidol concentrations, not by GC peak areas

**Fig. 3:**
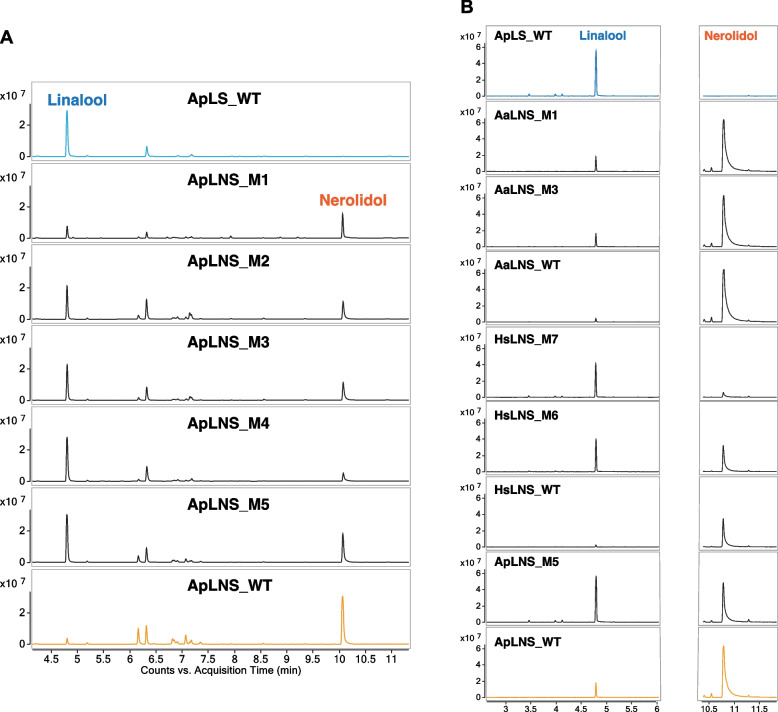
**A** The GC–MS chromatograms of ApLS, ApLNSs and its mutants (M1-M5). **B** The GC–MS chromatograms of ApLS, ApLNS, AaLNS, HsLNS and their mutants producing the highest amounts of linalool

As ApLNS shares the highest identify (78%, Fig. 1B) with ApLS, we started with ApLNS engineering using ApLS as the template. As shown in Fig. 2A and Fig. 3A, the combined mutations of S58A, M59L, I60V, T62P and V63L (ApLNS_M1) significantly decreased the nerolidol production by 77%, while the linalool production was increased marginally as compared to that of ApLNS wildtype (WT). Here, the yields of linalool and nerolidols of various enzymes in Fig. 2A were normalized by that of ApLNS WT, respectively. Further introduction of E64G to ApLNS_M1 (ApLNS_M2) greatly boosted linalool production to 245%. ApLNS_M3 (ApLNS_M2 with D67E) produced slightly higher linalool than ApLNS_M2, reaching 267%. Incorporating H68E into ApLNS_M3 (ApLNS_M4) led to a remarkable increase of 327% in linalool production. For ApLNS_M1/2/3/4, the nerolidol production was similar, 21–25% of that of ApLNS WT. For ApLNS_M5, we removed the S58A mutation from ApLNS_M4. ApLNS_M5 produced the highest linalool (~ 441%) among all the mutants, which was comparable to that of the highly active ApLS (425%). Interestingly, the nerolidol production of ApLNS_M5 (48%) was also the highest among all the mutants but lower than that of ApLNS WT. Therefore, although ApLNS_M5 produced the highest amount of linalool, the ApLNS_M4 had the highest linalool/nerolidol ratio (64%:36%) among all the mutants, and significantly higher than that of ApLNS WT (5%:95%, Fig. 2B and Fig. 3A). Further addition of the outside-the-loop-region mutations S43E, M50A or M88L to ApLNS_M5 (ApLNS_M6-8) had slightly negative effect on linalool and nerolidol production (Fig. 2 and Table 2). The results clearly indicated that loop region is critical in determining the substrate preference of ApLNS and ApLS. By mutating these amino acid residues, we could tailor ApLNS to favor GPP over FPP, thus producing more linalool than nerolidol. However, we could not eliminate the nerolidol production completely by mutating the region alone, indicating that there are other residues co-regulating the FPP substrate acceptance.

**Table 2:** Enzyme mutant information

No	Enzymes		Amino acid substitution	Production level (%)	
				Linalool	Nerolidol
1	ApLS WT			100%	0%
2	ApLNS	WT		5%	95%
3		M1	S58A, M59L, I60V, T62P, V63L	32%	68%
4		M2	S58A, M59L, I60V, T62P, V63L, E64G	55%	45%
5		M3	S58A, M59L, I60V, T62P, V63L, E64G, D67E	58%	42%
6		M4	S58A, M59L, I60V, T62P, V63L, E64G, D67E, H68E	64%	36%
7		M5	M59L, I60V, T62P, V63L, E64G, D67E, H68E	36%	64%
8		M6	S43E, M59L, I60V, T62P, V63L, E64G, D67E, H68E	34%	66%
9		M7	M50A, M59L, I60V, T62P, V63L, E64G, D67E, H68E	31%	69%
10		M8	M59L, I60V, T62P, V63L, E64G, D67E, H68E, M88L	28%	72%
11	GmLNS	WT		45%	55%
12		M1	S59A	52%	48%
13		M2	P65G, S66T, R67K, D68E, H69E	43%	57%
14	HsLNS	WT		2%	98%
15		M1	S59A	7%	93%
16		M2	F60L	7%	93%
17		M3	I61V	10%	90%
18		M4	I61V, E65G, S66T	10%	90%
19		M5	F60L, I61V, E65G, S66T	27%	73%
20		M6	F60L, I61V, E65G, S66T, H69E	49%	51%
21		M7	S59A, F60L, I61V, E65G, S66T, H69E	90%	10%
22		M8	S59A, F60L, I61V, E65G, S66T, H69E, R92A	94%	6%
23		M9	S59A, F60L, I61V, E65G, S66T, H69E, A182G	94%	6%
24		M10	S59A, F60L, I61V, E65G, S66T, H69E, R229Q	97%	3%
25		M11	S59A, F60L, I61V, E65G, S66T, H69E, Q317E	95%	5%
26	AaLNS	WT		4%	96%
27		M1	S73A	7%	93%
28		M2	M74L	1%	99%
29		M3	T75V	6%	94%
30		M4	S73A, T75V	1%	99%
31		M5	T75V, Y76G, H78L, H79G, N80T	No production detected	
32		M6	T75V, Y76G, H78L, H79G, N80T, F83E		
33	PcLNS-1			9%	91%
34	PcLNS-2			3%	97%

### Engineering the hCD loop of other LNSs for a higher activity of linalool production

**Fig. 4:**
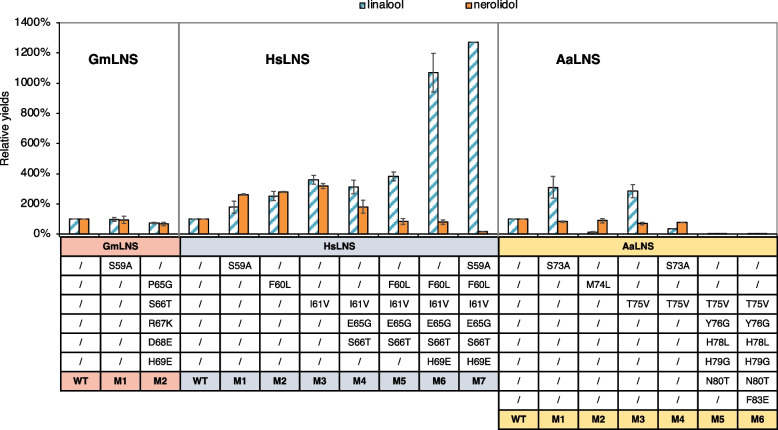
The effects of the loop region mutation on linalool and nerolidol production for GmLNS, HsLNS and AaLNS. The values are normalized to that of their own wildtype (WT). The standard deviation was calculated with three batches of GC–MS data

**Fig. 5:**
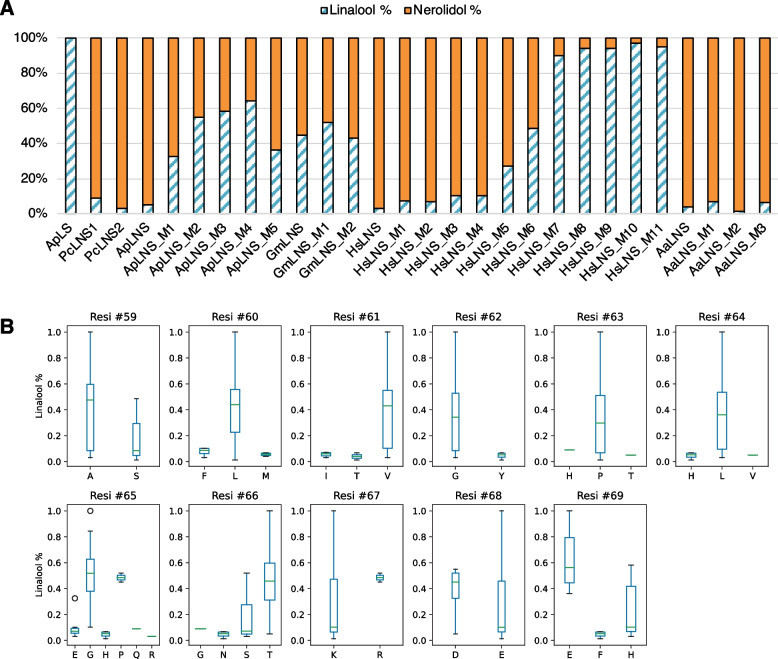
(**A**) Product distribution of linalool and nerolidol for various LNSs and their mutants. Here, the ratio was calculated by linalool and nerolidol concentrations, not by GC peak areas. (**B**) Statistical analysis of the effect of individual amino-acid-residue mutation on LNS selectivity. The residual position is referenced by that of ApLS

The loop mutation of ApLNS dramatically increased linalool production and simultaneously reduced nerolidol production. This inspired us to further evaluate the same loop region for other LNSs. For GmLNS, unlike ApLNS, the mutations (GmLNS _M1, S59A; GmLNS_M2, P65G-S66T-R67K-D68E-H69E) did not significantly changed linalool and nerolidol production, nor the ratio (Table 2, Fig. 4 and Fig. 5A), possibly because its wildtype already produces relatively high linalool/nerolidol ratio (45%:55%, Table 1, Table 2, and supplementary Fig. 2).

In contrast, the mutations in the loop region markedly enhanced the linalool production for HsLNS. The single amino acid mutations S59A (HsLNS_M1), F60L (HsLNS_M2) and I61V (HsLNS_M3) increased both linalool and nerolidol production as compared to the wildtype HsLNS. Specifically, the mutations S59A, F60L and I61V to HsLNS increased linalool yield to 179%, 252% and 361%, respectively, and boosted nerolidol yield to 261%, 281% and 318%, respectively, as compared that of the wildtype HsLNS (Fig. 4). However, the triple mutation I61V-E65E-S66T (HsLNS_M4) had slightly lower linalool production (312%) and lower nerolidol production (181%) than HsLNS_M3 with a single mutation. Introduction of F60L to HsLNS_M4 (HsLNS_M5) increased linalool production (383%) and further decreased nerolidol yield (83%). Incorporating H69E to HsLNS_M5 (HsLNS_M6) had a very significant boost on linalool production (1068%). Lastly, the S59A addition to HsLNS_M6 further increased linalool yield to 1272% and reduced nerolidol yield to 17% (Fig. 3B and Fig. 4). Therefore, through the loop region mutation, the linalool/nerolidol ratio increased gradually from 2%:98% in the wildtype HsLNS to 90%:10% in the HsLNS_M7 (Fig. 5A).

**Fig. 6:**
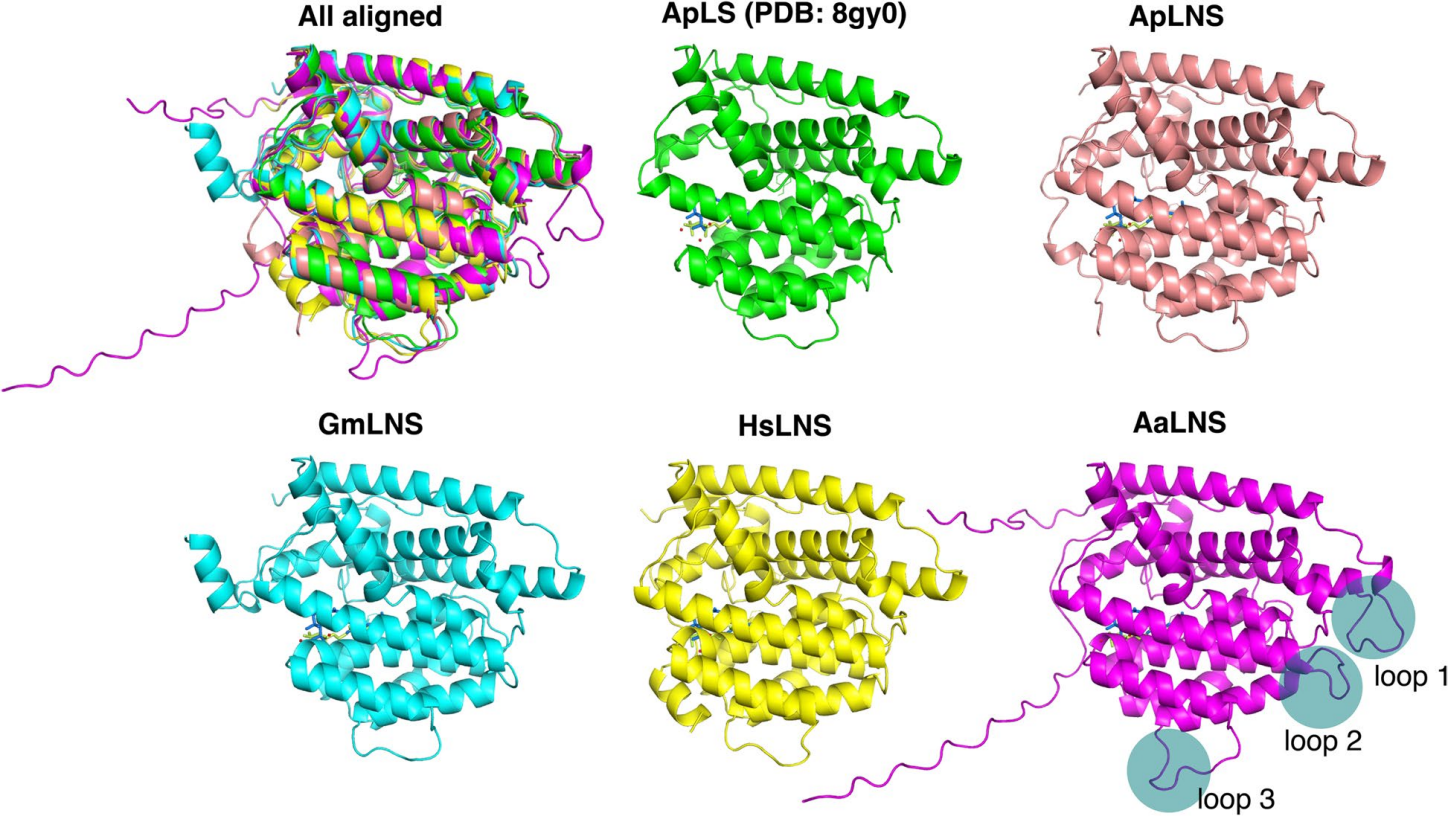
Crystal/AlphaFold2 structures of the five enzymes. ApLS is crystal structure (PDB accession is 8GY0): green. Except ApLS, the other structures are predicted by AlphaFold2. ApLNS: salmon; GmLNS, cyan; HsLNS, yellow; AaLNS, magenta

AaLNS sequence (Fig. 1) and structure are noticeably different from the other 4 enzymes, particularly for the loop regions (Fig. 6). While we mutated the amino acids in the loop region in AaLNS, S73A (AaLNS_M1) or T75V (AaLNS_M3) mutation increased linalool production to ~ 286–310% of wildtype AaLNS (Fig. 3B). In the meantime, nerolidol production was slightly reduced by about 20%. However, combining S73A with T75V (AaLNS_M4) resulted in 65% reduction in linalool production. M74L (AaLNS_M2) mutation reduced linalool production by 86% compared to that of wildtype (Fig. 4). Introducing multiple mutations like T75V-Y76G-H78L-H79G-N80T (AaLNS_M5) and T75V-Y76G-H78L-H79G-N80T-F83E (AaLNS_M6) inactivated the AaLNS, completely abolishing both linalool and nerolidol production (Fig. 4). For the functional mutants (M1-M3) of AaLNS, the linalool/nerolidol ratio varied, with M1 and M3 increased from 4% (WT) to 7% and but M2 decreased to 1% (Fig. 5A).

### Engineering residues surrounding conserved regions for HsLNS

Based on multiple protein sequence alignment (Supplementary Fig. 3), we identified four key residues present in ApLS that are noticeably different from the other LNSs. In brief, A92 is present near the ^84^DExxD^88^ motif; G181 is near the aspartate-rich motif; Q228 is present near NSD triad and E316 is adjacent to the ^302^WxxxxxRY^309^ motif. We hypothesized that these residues might play a role in determining the high selectivity of ApLS. To test this hypothesis, we individually introduced the four mutations (R92A, A181G, R229Q and Q316E) to HsLNS_M7, resulting in HsLNS_M8 to in HsLNS_M11. The mutation R229Q further increased the linalool/nerolidol ratio from 90%:10% (HsLNS_M7) to 97%:3% (HsLNS_M10). However, the new mutations could not completely eliminate nerolidol production (Fig. 5A).

### Structural understanding of the effect of mutations

We did a summary for the effects of all the mutants on the 4 LNSs. As shown in Fig. 5B. Most of the individual residue mutations have limited impacts on the overall selectivity of the enzymes, indicating the effects are likely attributed to the synergistic effect of multiple-residue mutation. Of note, some residues, such as the residue #65, show that LNSs with mutations to proline (Pro) and glycine (Gly) tends to produce linalool than nerolidol, compared with the mutations to arginine (Arg), histidine (His), Glutamic acid (Glu) or glutamine (Gln); for the residue #69, LNSs with mutation Glu to are more likely to produce more linalool compared to the mutations to phenylalanine (Phe) and His.

**Fig. 7:**
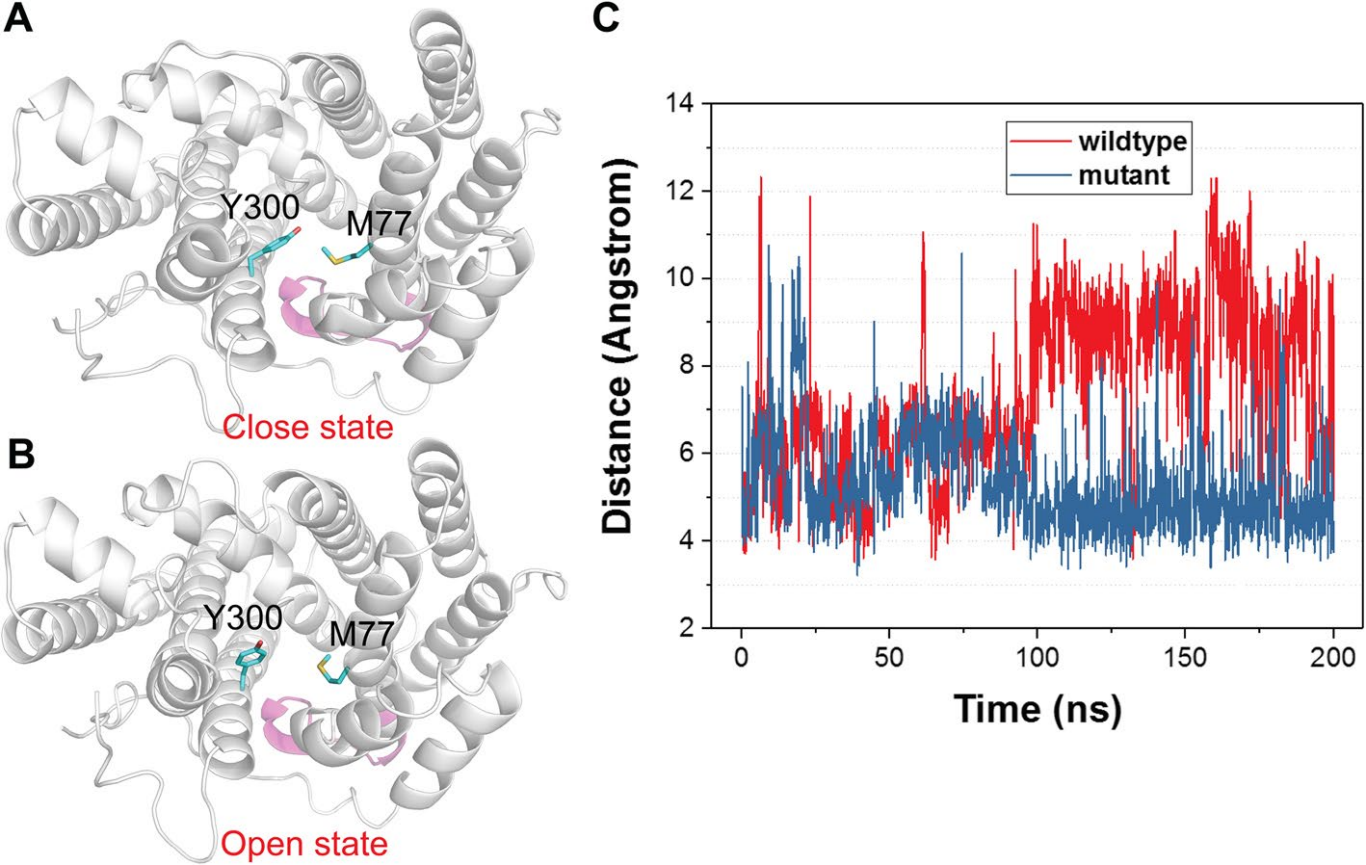
Molecular dynamics simulation results for HsLNS and HsLNS_M7. **A** Close stage, Y300 faces inward, closer to M77, to form a smaller binding pocket. **B** Open stage, Y300 stretches outward to form a large binding pocket. The hCD loop is highlighted in pink. **C** fluctuation of the distance between two gating residues (Y300 and M77) along the simulation trajectories

Next, we investigated the effect of the hCD loop mutation on other residues, particularly those active sites. According to Fig. 7, Y299 is located near the loop region and plays a key role in substrate selectivity of ApLS, as demonstrated in our previous work (T et al. 2023). The corresponding residues for Y299 in ApLS are Y299 in GmLNS, Y300 in HsLNS, Y298 in ApLS, and E300 in AaLNS. We hypothesized that the loop mutation might induce a conformational change in Y299 (to be simple, we use Y299 to represent the corresponding Tyr residues for all the enzymes), further influencing substrate preference. However, the computational modelling indicated that the Y299 orientation was not affected significantly for all the four LNSs by the loop mutation as shown in the Supplementary Fig. 4. Y299 in most of the mutants overlapped nicely with their respective wildtypes. In fact, we could not identify any residue in the active sites of the mutants that differs significantly from that in the wildtype. The results indicated that the hCD loop mutation might have a global effect on the active site, rather than targeting specific residues.

Therefore, we compared the molecular docking scores (or docking energy) for 4 LNSs and their respective mutants. As shown in Table 3, the FPP binding scores of ApLNS_M5 and HsLNS_M7 were markedly lower (~ 88%) as compared to that of their wildtypes. The results correlated nicely with the decreased nerolidol activity for these two mutants. As for AaLNS_M5 and GmLNS_M2, the binding scores were similar (~ 100%) to that of their wildtypes. The result was also consistent with the relatively similar activity of GmLNS_M2 to its wildtype. AaLNS_M5 was an outlier as it lost its activity completely. The GPP binding score of the mutants were similar (93–99%) to that of their wildtype, except for HsLNS_M7, which was 91% of its wildtype. Unlike the FPP binding score, the GPP binding score could not explain the increased linalool activity of the mutants. This could be because that the binding score only correlated with the affinity of an enzyme for its substrate like *K*_*m*_, while other important factors for enzyme activity, such as the stability of transition states and products, enzyme expression, as well as energetic barriers, could not be captured by docking scores.

**Table 3:** Molecular docking scores for Enzyme-FPP/GPP

Enzyme	FPP	GPP
AaLNS	55.90	39.85
ApLNS	48.37	39.62
GmLNS	43.30	42.25
HsLNS	49.06	45.46
AaLNS_M5	55.94	37.11
ApLNS_M5	42.58	37.23
GmLNS_M2	43.79	42.02
HsLNS_M7	43.30	41.54

To gain more insight, we also conducted molecular dynamics (MD) simulations for both apo HsLNS and apo HsLNS_M7, as described in the Methods section. Notably, the data in Fig. 7 revealed that in the apo wild-type HsLNS, Y300 has a higher tendency to extend outward (open stage), creating additional space to accommodate the longer FPP substrate. In contrast, together with M77, inward-facing Y300 (close state) in the HsLNS_M7 could prevent the FPP entering the substrate binding pockets. As such, the MD simulation provides a plausible explanation for the increased selectivity of the mutant toward linalool, while reducing its activity to nerolidol.

## Discussion

With over 100,000 molecules identified, terpenoids or isoprenoids are the largest family of natural products, represents a third of all the compounds described in the Dictionary of Natural Products (Whitehead et al. 2023). Terpene synthases are the primary contributor to the enormous diversity of terpenoids. Among them, many sesquiterpene synthases can also accept GPP to produce monoterpenes, like LNSs, Agr4 from *Cyclocybe aegerita* (Zhang et al. 2020) and a number of bacterial sesquiterpene synthases (Reddy et al. 2020). Similarly, some known sesquiterpene synthases can also accept geranylgeranyl diphosphate (GGPP) to produce diterpenes, like Agr2 from *C. aegerita* (Hoberg et al. 2024). Engineering such bifunctional enzymes to exclusive monofunctional ones is not a trivial study, but critical for applications in biosynthesis, biocatalysis, and industrial biotechnology.

Due to the structural difference of four LNSs, the hCD loop mutation has different effects on them. The loop mutation greatly increased the linalool production and reduced nerolidol production for ApLNS and HsLNS. In contrast, the selectivity of GmLNS was only slightly affected by the mutation. AaLNS is an outlier among the four LNSs with a few regions different from others. The combined loop mutation abolished AaLNS activity completely. Of note, none of the residues in the hCD loop directly interact with the substrate, but their combination impacts the binding of these enzymes to their substrates, particularly FPP. As such, rational design might not consider these mutations for improving substrate selectivity. Our study here is instrumental for future enzyme design efforts.

The loop mutation is essential for improving monoterpene activity, but itself is insufficient to eliminate the sesquiterpene activity. Other mutations like R229Q could further improve the substrate selectivity. With R229Q and the loop mutation, HsLNS_M10 mutant is very close (97%) to a monofunctional linalool synthase. Of note, previous efforts on engineering taxadiene synthase like Y89E variant resulted in higher specificity than the wildtype, however, at the cost of reduced activity (2.8% of wildtype) (Schrepfer et al. 2016). In contrast, our engineering effort here greatly improved the selectivity of LNSs, while maintaining similar catalytic activities. Previous and our studies here indicate that engineering substrate selectivity without compromising enzyme activity is a challenging task, even with the help of protein structure elucidation and molecular docking simulation.

According to the molecular docking simulation results for the wild types and their mutants, FPP with a longer tail appears to be more sensitive to the loop mutations. This is understandable, as FPP’s long tail must penetrate deeper into the pocket, and the loop mutation makes it more difficult to achieve. The computational results for FPP are comparable with their experimental activities in most cases. However, GPP has a shorter tail, and its binding scores are less affected by the remote loop mutations, which does not explain the experimental activities. Therefore, we postulate that other key factors, such as the stability of transition states and products, may have a more significant impact on the enzyme activity.

Based on the MD simulation results, two gating residues (Y300 and M77) are critical for the sesquiterpene synthase activity. In ApLS, the two residues block the FPP from entering the pocket. Similarly, in the mutants, Y300 tends to contact with M77 through favourable hydrophobic interactions, which may hinder the penetration of FPP's long tail; while in the wildtype, Y300 is more likely to be positioned outward, allowing for the formation of a deep pocket that can accommodate the long tail of FPP. Therefore, our simulations lead us to an intriguing hypothesis that the loop mutations affect substrate preferences, possibly by fine-tuning the interactions between the two gating residues in the delicate manner. This observation is instrumental for de novo design and engineering of bifunctional terpene synthases to exclusive monoterpene synthases.

## Materials and methods

### Bacterial strains and constructs

The seven fungal terpene synthases namely, ApLS, ApLNS, HsLNS, AaLNS, GmLNS, PcLNS-1 and PcLNS-2 were studied (details in Table 1). Their amino acid sequences were provided in Supplementary Notes. All the genes encoding these enzymes were codon-optimized and cloned into pET-11a plasmid fused with 6 histidine in the N-terminal as previously described (Zhang et al. 2021). Each of these served as the template for site-directed mutagenesis using iProof PCR kit (Biorad) and the primers listed in Supplementary Table 1.

Production strain was *E. coli* BL21 Gold DE3 strain (Stratagene), which carried the plasmid p15A-camR-T7-dxs-idi and the LS/LNS plasmids. The enzymes, DXS and IDI from *E. coli* were overexpressed to enhance the supply of terpene precursors (Zhang et al. 2020).

### Media and culture conditions

LB broth (10 g/L tryptone, 5 g/L yeast extract, 10 g/L NaCl) was used for all molecular cloning and pre-cultures. For production study, 1 × ZYM autoinduction media (10 g/L tryptone, 5 g/L yeast extract, 2 mM MgSO_4_, 5 g/L glycerol, 0.5 g/L glucose, 25 mM Na_2_HPO_4_, 25 mM KH_2_PO_4_, 50 mM NH_4_Cl, 5 mM Na_2_SO_4_ and 0.02 mM FeCl_3_, 15 mM lactose) was used (Studier 2005). In the initial stage, *E. coli* utilizes glucose as the main carbon source. Upon depletion of glucose, the cells start using lactose and glycerol. To maintain the two plasmids pET11a and p15C-SI, the culture media were supplemented with 100 ug/ml ampicillin and 34 ug/ml chloramphenicol respectively.

### SPME-GC/MS run and analysis

To detect the desired compounds, cells were grown in 4 ml of 1 × ZYM in 20 ml vials with headspace for 20–24 h at 28 °C, 300 rpm before Solid-phase microextraction (SPME)-GC/MS run. The compounds were initially sampled at 60 °C for 20 min with a DVB/CAR/PDMS (50/30 μm divinylbenzene/ carboxen/ polydimethylsiloxane) fiber (length 1 cm; Supelco, Steinheim, Germany). Subsequently, they were desorbed for 1 min in the split inlet (250 °C; SPME liner, 0.75 mm i.d.; Supelco) and analyzed by an Agilent 7980B GC equipped with an Agilent 5977B MSD. Samples were injected into Agilent DB5ms column with a split ratio of 40:1 at 240 °C. As for the oven program, it was initially set at 80 °C for 1 min, then raised up to 210 °C at 10 °C/min, then to 310 °C at 60 °C/min and finally maintained at 310 °C for another 2 min. Mass spectrometer was operated in EI mode with full scan analysis (m/z 33–300, 9 scans/s) (Zhang et al., 2021). In the end, the detected compounds were identified using respective chemical standards (Sigma-Aldrich, Singapore).

### Phylogenetic and alignment analysis

Full amino acid sequences were used to build a phylogenetic tree. Alignment and the phylogenetic analysis were carried out by the EMBL-EBI Job Dispatcher sequence analysis tools framework (Madeira et al. 2024). The job ID was clustalo-R20240824-024400–0117–11142682-p1m and can be retrieved by the website https://www.ebi.ac.uk/jdispatcher/. The framework embedded the Clustal Omega program version 1.2.4 and the Neighbour-Joining method without distance corrections. The percent identity matrix was also obtained by the EMBL-EBI framework with job ID of clustalo-R20240824-051415–0309–28760261-p1m.

### Computational modelling and analysis

Each enzyme was modelled using the following detailed protocol: (1) The initial structural model was predicted using the Modeller program, leveraging a high sequence identity with the crystal structure of linalool synthase (PDB: 8GY0). (2) Loops and sidechains were refined in the Sybyl-X package (v2.1), generating a diverse set of models. (3) The model with the lowest energy was selected for further optimization in an aqueous environment. (4) The selected model was solvated using the TIP3P water model and neutralized by adding counter ions with tleap. (5) The system was minimized in two stages: first, with restraints on the enzyme heavy atoms using a harmonic force constant of 1.0 kcal/mol/Å2 to minimize water molecules, and then fully minimized without any restraints to achieve system relaxation. (6) The system was gradually heated to 298.15 K over 25 ps under NVT conditions, with position restraints of 1.0 kcal/mol/Å2 applied to the enzyme heavy atoms. (7) Equilibration continued for another 500 ps in the NVT ensemble with position restraints of 1.0 kcal/mol/Å2 applied to the enzyme heavy atoms. (8) Three rounds of equilibration, each 2 ns in length, were performed in the NPT ensemble with decreasing harmonic restraints on the enzyme heavy atoms (1.0, 0.1, and 0.01 kcal/mol/Å2, respectively). (9) The production simulation was conducted over 200 ns without any restraints, at a constant pressure of 1 atm and temperature of 298.15 K, using a Monte Carlo barostat for pressure control and a Langevin thermostat for temperature control. The ff19SB force field parameters were applied (Tian et al. 2020), and long-range electrostatic interactions were calculated using the Smooth Particle Mesh Ewald (SPME) method (Essmann et al. 1995) with a cutoff of 9 Å. The entire simulation process was repeated two times, all within the AMBER22 molecular dynamics software package (Case et al. 2023). This computational protocol was also applied to model the loop mutant of HsLNS.

All enzyme complexes with GPP/FPP were modelled based on our previous complex model for ApLS-Mg-GPP/FPP, as detailed in our earlier work (T et al. 2023). Subsequently, all enzyme sidechains were fully optimized using the Sybyl-X package (v2.1), with restraints applied to the three Mg2⁺ ions and the phosphate group of GPP/FPP, ensuring consistency in the binding mode between the conserved Mg2⁺ ions and the phosphate group of GPP/FPP. Finally, GOLD software (v2018) was used to evaluate the binding score between each enzyme and GPP/FPP in rescore mode. GPP/FPP served as the reference ligand, with the binding site defined by a spherical radius of 10 Å around the reference ligand. The GoldScore function was used for scoring, and simplex minimization was applied prior to rescoring.

## Supplementary Information

Supplementary Material 1.

## Data Availability

The authors confirm that the data supporting the findings of this study are available within the article [and/or] its supplementary materials. All the data that supports the findings of this study are available in the article, Supplementary Information, or upon request from the corresponding author.
