# Supplementary material for: Unravelling the aromatic symphony: redirecting bifunctional mushroom synthases towards linalool monofunctionality
Source: Adv Biotechnol (Singap). 2025 Jan 13;3(1):3. doi: 10.1007/s44307-024-00056-2 (PMC11740858; doi:10.1007/s44307-024-00056-2)
Supplement: Supplementary file 1 — Supplementary Material 1. [file 44307_2024_56_MOESM1_ESM.pdf]

Unravelling the Aromatic Symphony: Redirecting Bifunctional Mushroom Synthases towards Linalool Monofunctionality

Rehka T[1], Xixian Chen[1], Congqiang Zhang[1]*

[1]*Singapore Institute of Food and Biotechnology Innovation (SIFBI), Agency for Science, Technology and Research (A*STAR), Singapore, Republic of Singapore.*

*To whom correspondence may be addressed.

Congqiang Zhang: *31 Biopolis Way, Level 6, Nanos building, Singapore 138669*;

Email: congqiang_zhang@sifbi.a-star.edu.sg; zcqsimon@outlook.com

Supplementary Figures

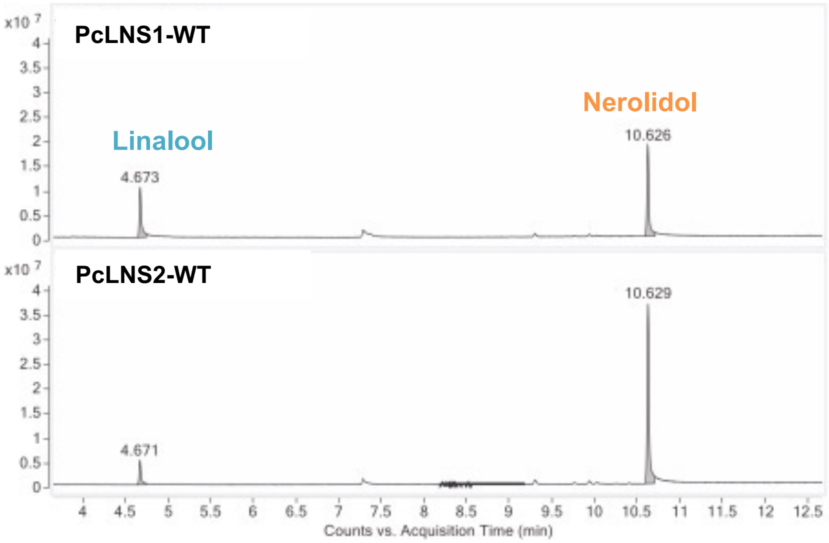

Supplementary Figure 1. The GC-MS chromograms of PcLNS.

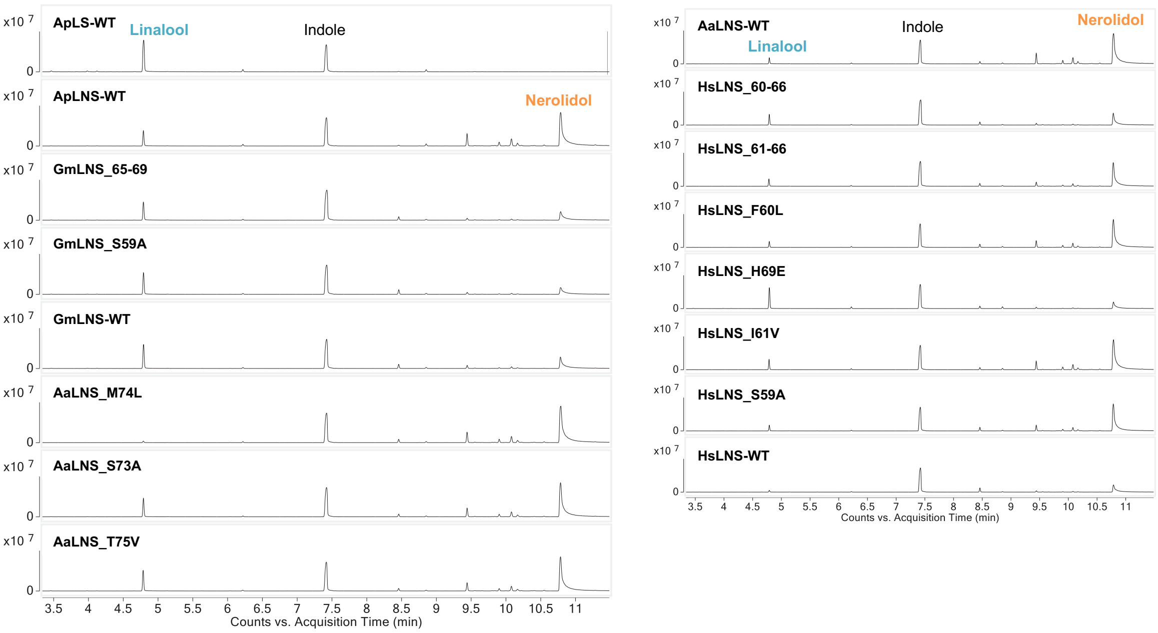

Supplementary Figure 2. The GC-MS chromograms of ApLS, ApLNS, GmLNS, AaLNS, HsLNS and their mutants.

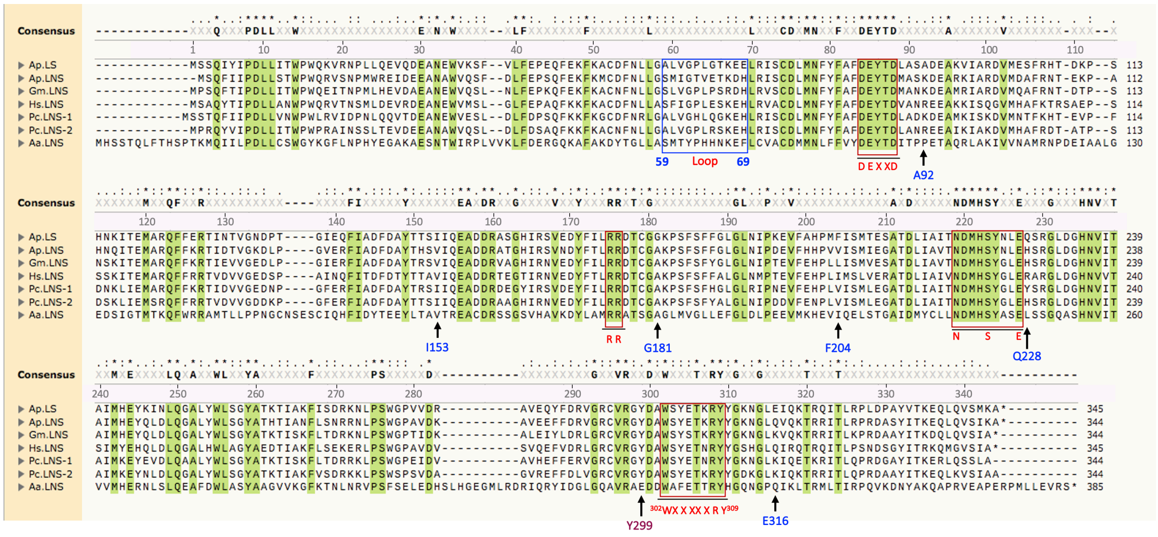

Supplementary Figure 3. Multiple protein alignment by Clustal Omega version 1.2.4. The consensus amino acids were highlighted in green. The loop region, DExxD, NSE and WxxxxRY motifs were highlighted in box.

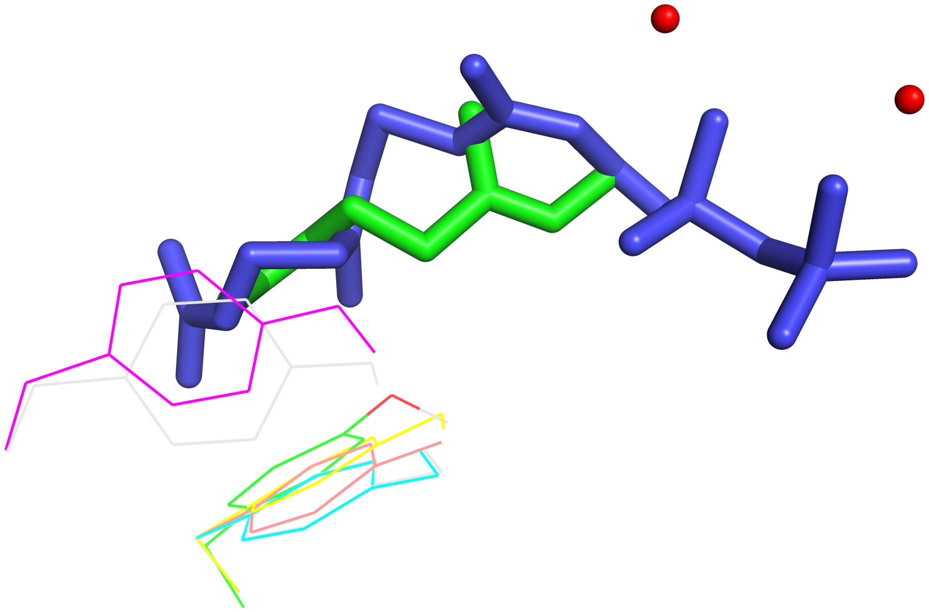

Supplementary Figure 4. Comparing the key residue (Y299 ApLS) between the wildtype and best mutants of LNSs with ApLS. The corresponding residues for Y299 in ApLS are Y299 in GmLNS, Y300 in HsLNS, Y298 in ApLS, and E300 in AaLNS. Colors are the same as Figure 6. ApLS: green. ApLNS: salmon; GmLNS, cyan; HsLNS, yellow; AaLNS, magenta; all the mutants: grey. FPP: blue sticks; GPP: green sticks; Mg^{2+}: red ball.

Supplementary Notes

Protein sequence

>AaLNS
MHSSTQLFTHSPTKMQIILPDLLCSWGYKGFLNPHYEGAKAESNTWIRPLVVKLFDERGQKAFAK
DYTGLLASMTYPHHNKEFLCVACDMMNLFFVYDEYTDITPPETAQRLAKIVVNAMRNPDEIAAL
GEDSIGTMTKQFWRRAMTLLPPNGCNSESCIQHFIDYTEEYLTAVTREACDRSSGSVHAVKDYLA
MRRATSGAGLMVGLLEFGLDLPEEVMKHEVIQELSTGAIDMYCLLNDMHSYASELSSGQASHNVI
TVVMHERNLSLQEAFDWLASYAAGVVKGFKTNLNRVPSFSELEDHSLHGEGMLRDRIQRYIDGL
GQAVRAEDDWAFETTRYHGQNGPQIKLTRMLTIRPQVKDNYAKQAPRVEAPERPMLLEVRS
>ApLS
MSSQIYIPDLLITWPWQKVRNPLLQEVQDEANEWVKSFVLFEPEQFEKFKACDFNLLGALVGPLGT
KEELRISCDLMNFYFAFDEYTDLASADEAKVIARDVMESFRHTDKPSHNKITEMARQFFERTINTV
GNDPTGIEQFIADFDAYTTSIIQEADDRASGHIRSVEDYFILRRDTCGGKPSFSFFGLGLNIPKEVFAH
PMFISMTESATDLIAITNDMHSYNLEQSRGLDGHNVITAIMHEYKINLQGALYWLSGYATKTIAKFI
SDRKNLPSWGPVVDRAVEQYFDRVGRCVRGYDAWSYETKRYYGKNGLEIQKTRQITLRPLDPAY
VTKEQLQVSMKA
>ApLNS
MSQFIIPDLLSTWPWQRVSNPMWREIDEEANAWVQSFDLFEPSQFEKFKMCDFNLLGSMIGTVET
KDHLRISCDLMNFYFAFDEYTDMASKDEARKIARDVMDAFRNTEKPAHNQITEMARQFFKRTIDT
VGKDLPGVERFIADFDAYTHSVIQEADDRATGHIRSVNDYFTLRRDTCGAKPSFSFFGLGLNIPDEV
FHHPVVISMIEGATDLIAVTNDMHSYNLEHSRGLDGHNVITAIMHEYQLDLQGALYWLSGYATHT
IANFLSNRRNLPSWGPAVDKAVEEFFDRVGRCVRGYDAWSYETKRYYGKNGLQVQKTRRITLRP
RDASYITKEQLQVSIKA
>GmLNS
MPSQFTIPDLLITWPWQEITNPMLHEVDAEANEWVQSLNLFEPKQFEKFKACNFNLLGSLVGPLPS
RDHLRVSCDLMNFYFAFDEYTDMANKDEAMRIARDVMQAFRNTDTPSNSKITEMARQFFKRTIE
VVGEDLPGIERFIADFDAYTRSVIQEADDRVAGHIRNVEDYFILRRDTCGAKPSFSFYGLGLNIPTE

VFEHPLLISMVESATDLIAVTNDMHSYGLEHSRGLDGHNVITAIMHEYQLDLQGALYWLSGYATKTISKFLTDRKNLPSWGPTIDKALEIYLDRLGRCVRGYDAWSYSTKRYYGKNGLKVQKTRRITLKPRDAAYITKDQLQVSIA

>HsLNS

MSAQYTIPDLLANWPWQRVTNSMLDEVRDEANEWVMSLGLFEPAQFKKFKACDFNLLASFIGPLESKEHLRVACDLMNFYFAFDEYTDVANREEAKKISQGVMHAFKTRSAEPSSSKITEMARQFFRRTVDVVGEDSPAINQFITDFDTYTTAVIQEADDRTEGTIRNVEDYFTLRRDTCGAKPSFSFFALGLNMPTEVFEHPLIMSLVERATDLIAIVNDMHSYGLERARGLDGHNVVTSIMYEHQLDLQGALHWLAGYAEDTIAKFLSEKERLPSWGPAVDVSVQEFVDRLGRCVRGYDAWSYETNRYYGSHGLQIRQTRQITLPSNDSGYITRKQMGVSIA

>PcLNS-1

MSSTQFIIPDLLVNWPWLRVIDPNLQQVTDEANEWVESLDLFDPSQFKKFKGCDFNRLGALVGHLQGKEHLRISCDLMNFYFAFDEYTDLADKDEAMKISKDVMNTFKHTEVPFDNKLIEMARQFFKRTIDVVGEDNPGFERFIADFDAYTRSIIQEADDRDEGYIRSVDEYFILRRDTCGAKPSFSFHGLGLRIPNEVFEHPLVISMLEGATDLIAITNDMHSYGLEYSRGLDGHNVITAIMKEYEVDLQAALYWLSGYATKTISKFLTDRRKLPSWGPEIDVAVHEFFERVGRCVRGYDAWSYETNRYYGKNGLKIQETKRITLQPRDGAYITKERLQSSLA

>PcLNS-2

MPRQYVIPDLLITWPWPRAINSSLTEVDEEANAWVQSLDLFDSAQFKKFKACNFNLLGALVGPLRSKEHLRISCDLMNFYFAFDEYTDLANREEAIKIAKDVMHAFRDTATPSDSKLIEMSRQFFRRTVDVVGDDKPGFERFIADFDAYTTSIIQEADDRAAGHIRNVEDYFILRRDTCGAKPSFSFYALGLNIPDDVFENPLVISMLEGATDLIAITNDMHSYGLEHSRGLDGHNVITAIMKEYNLDLQGALYWLSGYATKTIAKFVSDRKKLPSWSPSVDAGVREFFDLVGRCVRGYDAWSYETKRYYGDKGLKIQKTRRITLQPRDAAYITKEQLKVSIAA

Supplementary Tables

Table 1. Primers used in this study.

No.	Template	Primer name	Primer sequence	Remarks
1	pET11a-ApLNS_58-64	58-63.r	gtgatccttggtttccagcggaccaaccag	58-63.f same as 58-64.f
2	pET11a-ApLNS_58-68	58-64.f	accaaggatcacctgcgcatcagttgcgac	
3		58-64.r	caggtgatccttggtacccagcgga	
4		58-67.f	ggaacacctgcgcatcagttgcgac	
5		58-67.r	cgcaggtgttccttggtacccagc	
6	pET11a-ApLNS_59-68	S58A.f	gggcgcgctggttggtccgctgggt	
7		S58A.r	accagcgcgcccagcaggttgaaatcacaca	
8		S43E.f	gaaccggaacagttcgagaaattcaaggcgtg	
9		S43E.r	ctgttccggttcaaacaggtcgaaagac	
10		M50A.f	aaggcgtgtgatttcaacctgct	
11		M50A.r	atcacacgccttgaatttctcgaactggg	
12		M88L.f	gacctggcatccaaagacgaagcgcgt	
13		M88L.r	ggatgccaggtccgtgtattcgtcgaatgc	
14	pET11a-HsLNS	hyp1_S59A.f	ctggcagcctttattggtccgctggaa	
15		hyp1_S59A.r	aaaggctgccagaaggttgaagtc	
16		hyp1_F60L.f	gcaagccttattggtccgctggaaagt	
17		hyp1_F60L.r	aataaggcttgccagaaggttgaa	
18		hyp1_I61V.f	agctttgttggtccgctggaaagtaaa	
19		hyp1_I61V.r	accaacaaagcttgccagaaggtt	
20	pET11a-HsLNS_I61V	hyp1_61-66_FP	ggtccgctgggaactaaagaacaccttcgtgtt	
21		hyp1_61-66_RP	agttcccagcggaccaacaaagcttgc	

22		hyp1_60-66_RP	agttcccagcggaccaacaaggcttgccagaaggtt	hyp1_60-66_FP same as hyp1_61-66_FP
23	pET11a-HsLNS_60-66	hyp1_60-66_H69E_FP	aaagaagagcttcgtgttgcttgcgatctgat	
24		hyp1_60-66_H69E_RP	acgaagctcttctttagttcccagcggacca	
25	pET11a-HsLNS_60-69	hyp1_59-69_FP	ctggcagcccttgttggtccgctgggaact	
26		hyp1_59-69_RP	accaacaagggctgccagaaggttgaagtc	
27	pET11a-AaLNS	9435_S73A.f	ctggcggctatgacttacccgcacca	
28		9435_S73A.r	catagccgccagcagaccggtgta	
29		9435_M74L.f	gcgtctctgacttacccgcaccacaac	
30		9435_M74L.r	agtcagagacgccagcagaccgg	
31		9435_T75V.f	tctatggtttacccgcaccacaacaaa	
32		9435_T75V.r	gtaaaccatagacgccagcagacc	
33	pET11a-AaLNS_S73A	9435_73-75_FP	gcggctatggtttacccgcaccacaacaaa	
34		9435_73-75_RP	cgggtaaaccatagccgccagcagaccggt	
35	pET11a-AaLNS_T75V	9435_75-80_FP	gttggcccgctcggcaccaaagaattcctgtgtgtg	
36		9435_75-80_RP	ggtgccgagcgggccaaccatagacgccagcag	
37	pET11a-AaLNS_75-80	9435_75-80_F83E_FP	aaagaagaactgtgtgtggcttgcgatatga	
38		9435_75-80_F83E_RP	cacacagttcttctttggtgccgagcgggc	
39	pET11a-GmLNS	gal2_S59A.f	ctgggcgccctggttggaccgctgcca	
40		gal2_S59A.r	cagggcgcccagcaggttgaagtt	
41		gal2_65-69_FP	ctgggaaccaaggaagagcttcgtgtgtcctgcga	
42		gal2_65-69_RP	ctcttccttggttcccagcggtccaaccaggga	
